# Eastern Mediterranean region tuberculosis economic burden in 2014

**DOI:** 10.1186/2047-2994-4-S1-P102

**Published:** 2015-06-16

**Authors:** A Sargazi, Z Sepehri, A Sagazi, PK Nadakkavukaran Jim, Z Kiani

**Affiliations:** 1 Medical faculty, Zabol University of Medical Sciences, Zabol, Islamic Republic Of Iran; 2 Research department, Zabol University of Medical Sciences, Zabol, Islamic Republic Of Iran; 3 Civil faculty, Sistan and Baloochestan University, Zahedan, Islamic Republic Of Iran; 4 Oracle e-Business Department, Tech Mahindra, Linlithgow, UK; 5 Medical faculty, Kerman medical university, Kerman, Islamic Republic Of Iran

## Introduction

Tuberculosis (TB) is known one of reasons of human mortality to this date cause by *Mycobacterium tuberculosis.*

## Objectives

In this study we tried to evaluate the direct economic burden of tuberculosis in the Eastern Mediterranean region in 2014 and determine its economic effect in the Gulf region.

## Methods

In this descriptive analytical study, data gathered from World Health Organization global report and World Bank data sheet. We considered decrease of disability-adjusted life year (DALY index) related to TB and its influence on gross domestic production (GDP) as TB economic burden. Data analyzed using SPSS software.

## Results

GDP per capita was $665, $24689, $1668, $3314, $4763, $6862, $5214, $52197, $9928, $11965, $3093, $21929, $1275, $93714, $25962, $1753, $4317, $43049, $1773 and $0 USD for Afghanistan, Bahrain, Djibouti, Egypt, Iran, Iraq, Jordan, Kuwait, Lebanon, Libya, Morocco, Oman, Pakistan, Qatar, Saudi Arabia, Sudan, Tunisia, United Arab Emirates, Yemen and Syria respectively in 2013. Eastern Mediterranean Mean GDP per capita was about 16744 USD in 2013. Gross GDP was 3% in Middle East in 2014, therefore Mean Eastern Mediterranean GDP per capita is calculated as 17246 USD for 2014.

Mean years of life expectancy at birth was 68 years, Tuberculosis weight was about 0.271 and TB incidence is considered in one year period. Mean years of life lost were 61 and 34 years for a child and an adult respectively. Number of TB infected cases was 750,000 and YLL, YLD and DALY is calculated as 5,138,000, 203,250 and 5,341,250 years. Economic burden related to TB mortality and morbidity is calculated more than 92 billion dollars in Eastern Mediterranean region in 2014.

## Conclusion

In this project we evaluated tuberculosis direct economic burden while indirect burden sometimes has more cost. The total money spent in tuberculosis care system was less than 250 million USD in Eastern Mediterranean region in 2014. According to TB high economic burden, We suggest to specialize sufficient budget for TB control in this region.

## Disclosure of interest

None declared.
